# IQGAP1 Interacts with Components of the Slit Diaphragm Complex in Podocytes and Is Involved in Podocyte Migration and Permeability *In Vitro*

**DOI:** 10.1371/journal.pone.0037695

**Published:** 2012-05-25

**Authors:** Claire Rigothier, Patrick Auguste, Gavin I. Welsh, Sébastien Lepreux, Colette Deminière, Peter W. Mathieson, Moin A. Saleem, Jean Ripoche, Christian Combe

**Affiliations:** 1 INSERM U1026, Université Bordeaux Segalen, Bordeaux, France; 2 Academic and Children’s Renal Unit, University of Bristol, Southmead Hospital, Bristol, United Kingdom; 3 Service de Néphrologie, Transplantation, Dialyse, Centre Hospitalier Universitaire, Bordeaux, France; 4 Université Bordeaux I, Talence, France; 5 Service d’Anatomie Pathologique, Centre Hospitalier Universitaire, Bordeaux, France; Institut National de la Santé et de la Recherche Médicale, France

## Abstract

IQGAP1 is a scaffold protein that interacts with proteins of the cytoskeleton and the intercellular adhesion complex. In podocytes, IQGAP1 is associated with nephrin in the glomerular slit diaphragm (SD) complex, but its role remains ill-defined. In this work, we investigated the interaction of IQGAP1 with the cytoskeleton and SD proteins in podocytes in culture, and its role in podocyte migration and permeability. Expression, localization, and interactions between IQGAP1 and SD or cytoskeletal proteins were determined in cultured human podocytes by Western blot (WB), immunocytolocalization (IC), immunoprecipitation (IP), and In situ Proximity Ligation assay (IsPL). Involvement of IQGAP1 in migration and permeability was also assessed. IQGAP1 expression in normal kidney biopsies was studied by immunohistochemistry. IQGAP1 expression by podocytes increased during their *in vitro* differentiation. IC, IP, and IsPL experiments showed colocalizations and/or interactions between IQGAP1 and SD proteins (nephrin, MAGI-1, CD2AP, NCK 1/2, podocin), podocalyxin, and cytoskeletal proteins (α-actinin-4). IQGAP1 silencing decreased podocyte migration and increased the permeability of a podocyte layer. Immunohistochemistry on normal human kidney confirmed IQGAP1 expression in podocytes and distal tubular epithelial cells and also showed an expression in glomerular parietal epithelial cells. In summary, our results suggest that IQGAP1, through its interaction with components of SD and cytoskeletal proteins, is involved in podocyte barrier properties.

## Introduction

Podocytes are epithelial cells with long foot processes that interdigitate around glomerular capillaries. A specialized protein complex junction between foot processes forms the slit diaphragm which is crucial for glomerular integrity. Nephrin is the major structural component of the slit diaphragm [1]. Other proteins such as podocin, phospholipase Cε1, CD2AP, α-actinin-4, CLIC5 and the scaffold proteins NCK 1 and 2 are also important for maintaining the slit diaphragm’s integrity. Defects in the expression of one of these proteins induce proteinuria resulting from effacement of foot processes and loss of glomerular barrier integrity [2], [3], [4], [5], [6].

IQGAP1, a scaffold protein involved in actin remodelling has been identified as being associated with nephrin in podocyte foot processes [7], [8]. In humans, IQGAP1 is a 189 kDa protein [9], localized in the cytoplasm and also associated with the cell membrane [7], [8]. IQGAP1 consists of several interaction domains: a calponin homology domain which binds filamentous (F)-actin, a WW domain interacting with ERK1-2 [10], an IQ domain interacting with calmodulin [11] and MEK1-2 [12], and a GRD domain (a GAP related domain, without GTPase activity) interacting with the Rho GTPases, Cdc42 and Rac1 [13], [14], [15]. The C-terminus domain interacts with CLIP170 [16], APC [17], β-catenin [18] and E-cadherin [19], [20].

By interacting with actin and the Rho GTPases Cdc42 and Rac1, IQGAP1 contributes to the actin network formation [21], [22], [23]. Moreover, IQGAP1 connects the actin network to microtubules by binding to APC and CLIP170 [16], [17]. IQGAP1 can also interact with E- and VE-cadherin [24] and β-catenin at the cellular junctions of epithelial and endothelial cells. By interacting with these different proteins, IQGAP1 plays a major role in cellular migration, cytoskeleton organization and linkage between actin cytoskeleton and microtubules.

Apart from being associated with nephrin in the proximity of the glomerular slit diaphragm in human and rats [7], [8], IQGAP1 is also present in migrating junctional complexes during rat glomeruli development, with different cell localizations over time: IQGAP1 is detected at cell-cell junctions between the earliest forming podocytes and migrates with the junctional complexes of differentiating podocytes. In the late capillary loop stage, IQGAP1 accumulates in foot processes where it colocalizes with podocalyxin [8].

**Figure 1:**
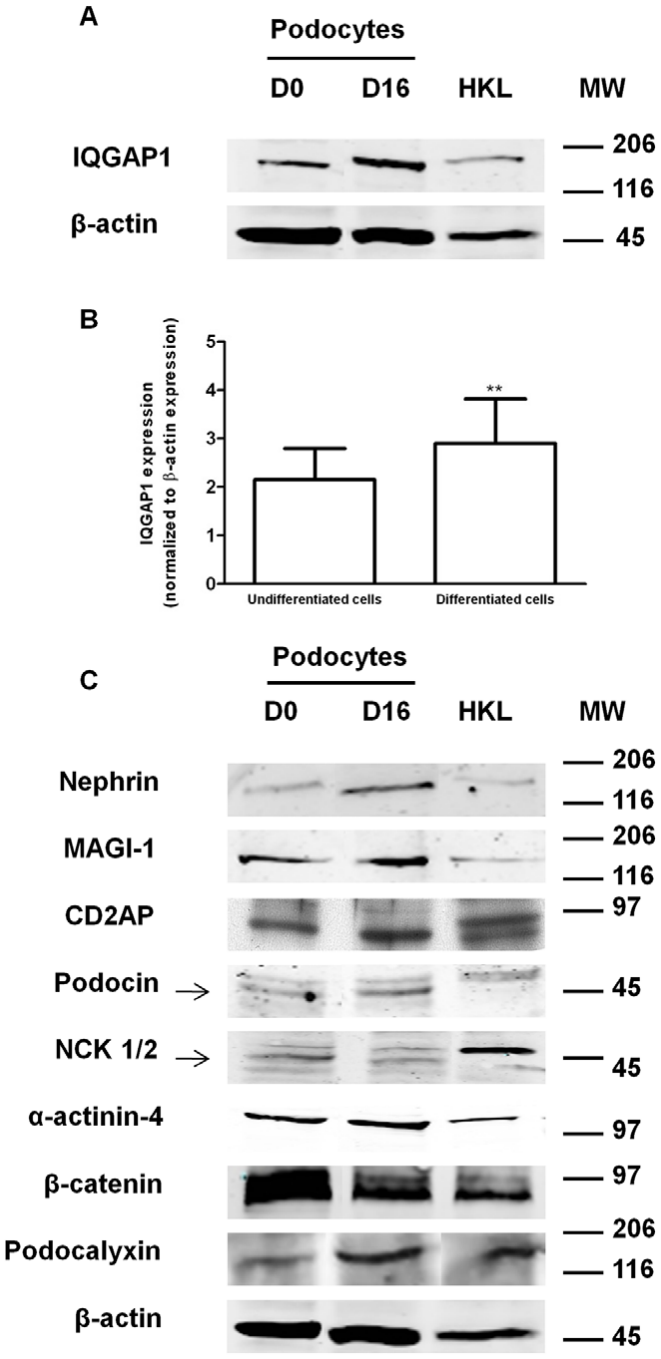
Western blot analysis of IQGAP1 expression in cultured podocyte extracts. (A) Western blot analysis of IQGAP1, detected at a molecular weight of 180 kD, and β-actin expressions during podocyte differentiation (top panel). Protein extracts were from undifferentiated (permissive temperature of 33°C, Day 0 (D0)) and differentiated (non-permissive temperature of 37°C, Day 16 (D16)) cultured immortalized podocytes. HKL: Human Kidney Lysate; MW: Molecular weight (kDa). The blot is representative of six independent experiments. (B) IQGAP1 expression at undifferentiated and differentiated stages. IQGAP1 expression, determined by densitometry, was plotted and reported to β-actin expression. Data are representative of six independent experiments. *Asterisk,* significantly different from undifferentiated cells (p<0.01, Wilcoxon’s test). (C) Western blot analysis of nephrin, MAGI-1, CD2AP, podocin, NCK 1/2, α-actinin-4, β-catenin, podocalyxin and β-actin during podocyte differentiation.

In this study, we have analyzed the protein partners of IQGAP1 in human cultured podocytes but also IQGAP1 involvement in podocyte migration and permeability, as well as IQGAP1 localization and expression on human normal kidney tissue sections.

**Figure 2:**
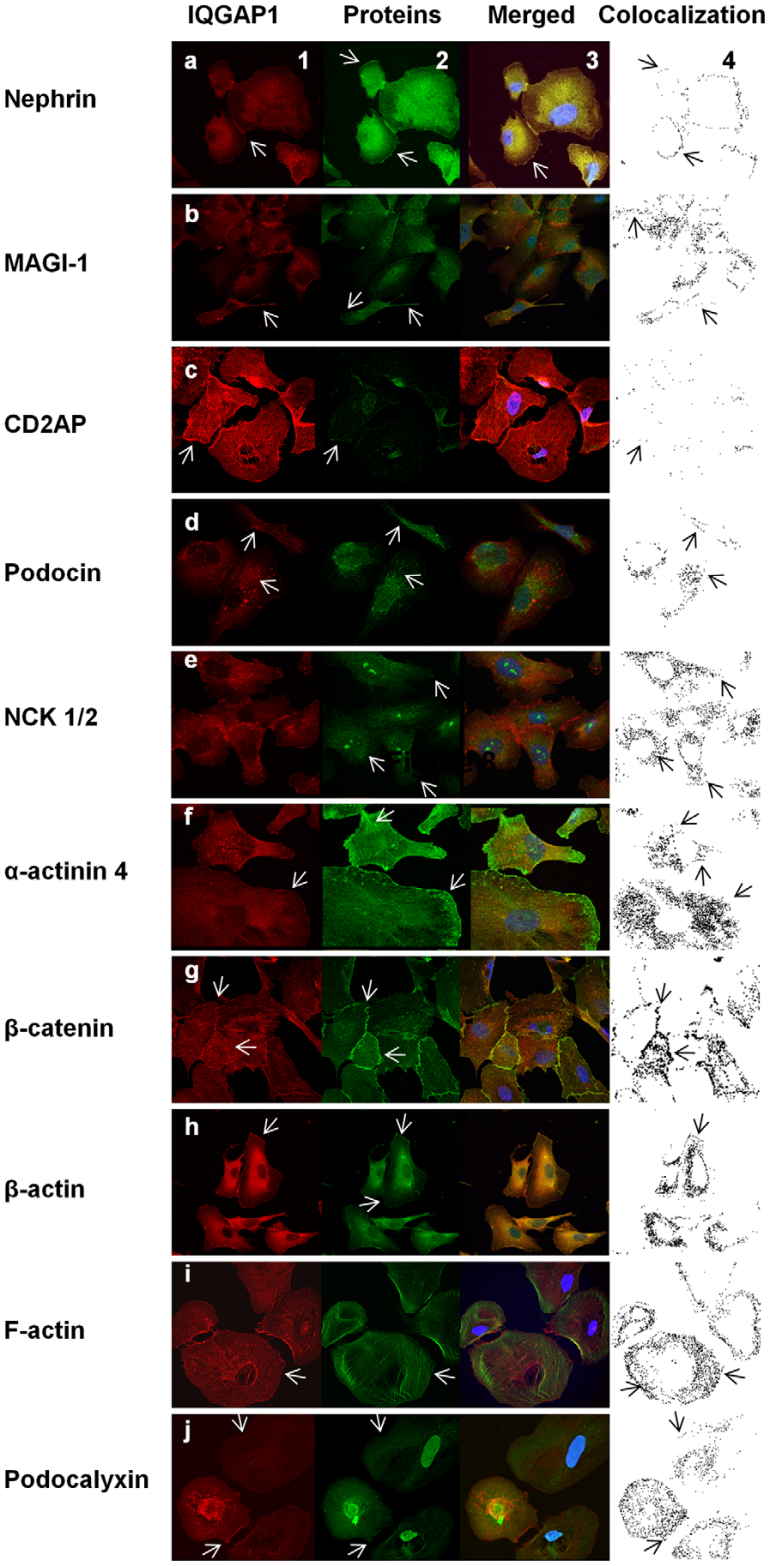
Subcellular localization of IQGAP1 and slit diaphragm proteins or podocalyxin. Double immunolabelling experiments were performed on cultured differentiated podocytes, and analyzed by confocal microscopy. Column 1: IQGAP1 (red). Column 2: nephrin (a), MAGI-1 (b), CD2AP (c), podocin (d), NCK 1/2 (e), α -actinin-4 (f), β-catenin (g), β-actin (h), F-actin (i) and podocalyxin (j) (green). Column 3: Colocalization of IQGAP1 and studied proteins, observed as a merge signal in yellow staining or as colocalization points in black color (Column 4) with the Leica analysis software.

## Results

### Expression of IQGAP1 in Human Conditionally Immortalized Podocytes

To study IQGAP1 expression in podocytes, we performed Western blot experiments. Cells were initially grown at the permissive temperature of 33°C - day 0 (D0) -, and were incubated up to 16 days (D16) at the non permissive temperature of 37°C (Details on morphological differences are available in Figure S1) [25], [26]. IQGAP1 protein was expressed in undifferentiated cells and its expression level increased, significantly in differentiated cells (p<0.01, n = 6) (Figure 1A and 1B). The expression of podocyte markers, nephrin, podocin, and podocalyxin also tended to increase during differentiation [25] (Figure 1C). Results are representative of different experiments (n = 6) and different human kidney lysates (Figure S2).

**Figure 3:**
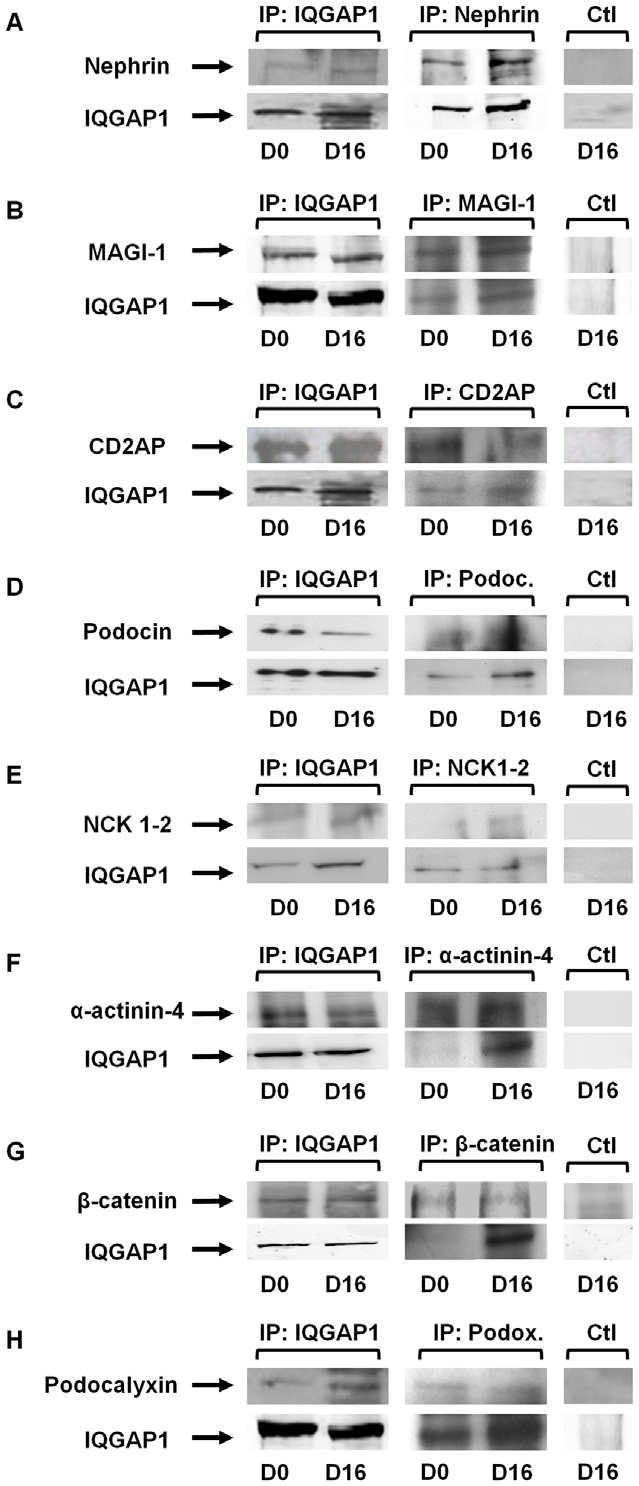
IQGAP1 interactions with proteins of the slit diaphragm and podocalyxin. IQGAP1 immunoprecipitation experiments were performed with undifferentiated podocytes (D0, 33°C) and differentiated podocytes (D16, 37°C), using equal amounts of total protein. The irrelevant antibody against tristetraprolin was used in control immunoprecipitations (Ctl). (A) Interaction between IQGAP1 and nephrin (data are representative of 6 independent experiments). (B to H) Interactions between IQGAP1 and MAGI-1, CD2AP, podocin, NCK1/2, α-actinin-4, β-catenin and podocalyxin (Data are representative of 5 independent experiments, 6 for podocalyxin).

### Colocalization of IQGAP1 with Proteins of the Slit Diaphragm

Immunofluorescence experiments were used to study the subcellular localization of the above proteins in differentiated podocytes. IQGAP1 was found in the perinuclear area, in cell extensions and associated to the plasma membrane with a stronger intensity on podocyte junctions (Figure 2, panel 1a to 1j). IQGAP1 perinuclear labelling, as for other studied proteins (nephrin, MAGI-1, NCK1 or/and 2), probably corresponded to endoplasmic reticulum and Golgi apparatus localization. Nephrin was expressed at cell membranes, particularly in podocyte junctions (Figure 2a, panel 2, arrows) and colocalized with IQGAP1 (Figure 2a, panels 3 and 4, arrows). MAGI-1 was localized at cell membrane and cytoplasmic extensions. Colocalization with IQGAP1 was mainly found in cytoplasmic extensions (Figure 2b, panel 1-4, arrows). CD2AP and podocin was only hardly expressed at the cell membrane, with a discrete colocalization with IQGAP1 (Figure 2c and 2d, panel 1-4, arrows). NCK 1 or/and 2 are scaffold proteins associated with nephrin [6]. In cultured podocytes, NCK 1 or/and 2 were found in the nucleus and at the cell membrane. A weak colocalization of NCK 1/2 with IQGAP1 was observed at the cell membrane (Figure 2e, panel 1-4). The α-actinin-4 labelling was found essentially with a filamentous distribution, evoking association with actin cytoskeleton, and associated with the cell membrane. Colocalization with IQGAP1 was preponderant in the cytoplasm and along the cell membrane (Figure 2f, panel 1-4, arrows). β-catenin had a similar localization pattern and colocalized with IQGAP1 at cell-cell contacts (Figure 2g, panel 1-4, arrows). β-actin was found in cytoplasm and associated with the plasma membrane. β-actin colocalized with IQGAP1 in both sites (Figure 2h, panel 1-4, arrows). The pattern of filamentous actin localization was similar to β-actin in podocytes. IQGAP1 and F-actin predominantly colocalized at the plasma membrane and on actin fibers to a less extent (Figure 2i, panel 1-4, arrows). As for CD2AP, podocalyxin was faintly expressed at the podocyte membrane where a detectable colocalization with IQGAP1 was found (Figure 2j, panel 1–4, arrows).

**Figure 4:**
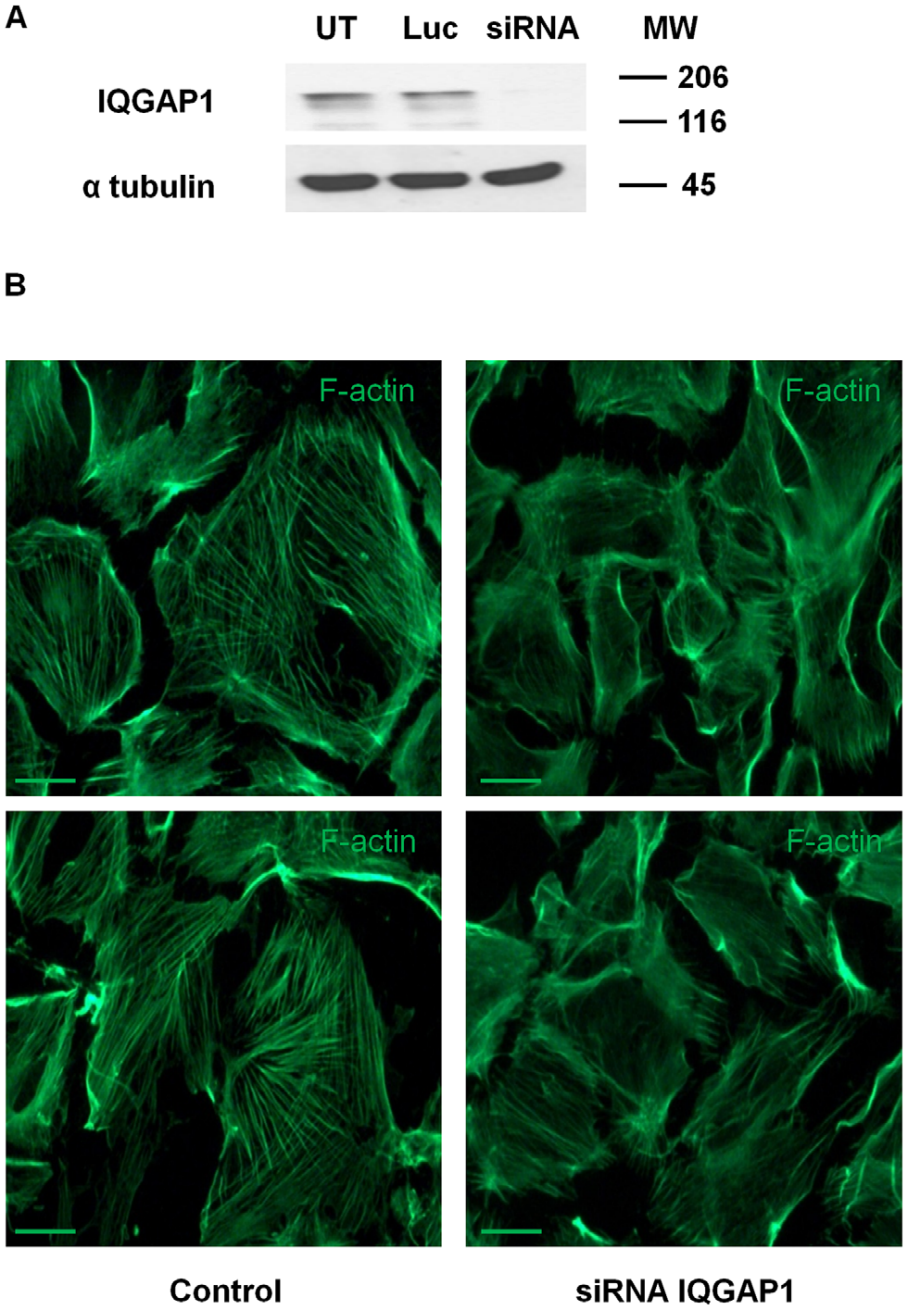
Effect of the down regulation of IQGAP1 expression on F- actin network. (A) IQGAP1 Western blot analysis showing IQGAP1 expression in podocytes transfected with siRNA IQGAP1 as compared with untransfected podocytes (UT) or podocytes transfected with siRNA directed at luciferase (Luc). (B) F-actin labelling after siRNA IQGAP1 transfection in comparison to control cells (untransfected cells). Scale bars, 50 µm.

**Figure 5:**
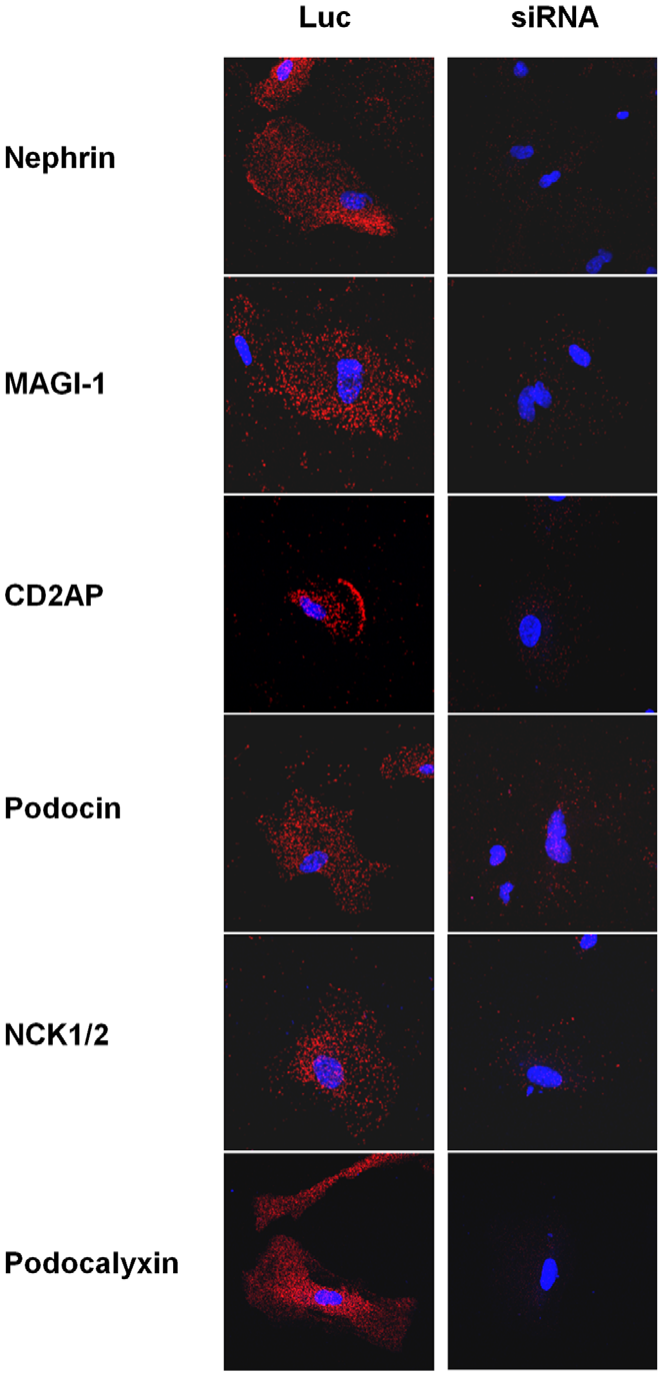
Cellular localization of interactions between IQGAP1 and different proteins of the slit diaphragm: In situ Proximity Ligation assay. In situ Proximity Ligation assays confirmed in cells the interactions between IQGAP1 and nephrin, MAGI-1, CD2AP, podocin, NCK1/2 and podocalyxin. Cells were transfected with siRNA Luc (Luc) or siRNA IQGAP1 (siRNA).

**Table 1:** siRNA used in the study.

siRNA	Effect	Sequence
siRNA 672	Inhibition	UGAAGCUAUUGACCGUAGA[TT]
siRNA 3014	Inhibition	UCUUCACACUCUACAACUA [TT]
siRNA Luc	Control	CGUACGCGGAAUACUUCGA[TT]

### Interaction Between IQGAP1 and Nephrin, MAGI-1, CD2AP, Podocin, NCK1 or/and 2, α-actinin-4, β-catenin and Podocalyxin

In order to characterize IQGAP1 protein interactions in undifferentiated and differentiated podocytes, IQGAP1 immunoprecipitation experiments were performed using equal amounts of total protein, associated with reverse immunoprecipitations. We first confirmed the interaction between IQGAP1 and nephrin [7], [8], and showed that this interaction occurred both in undifferentiated and differentiated cells (Figure 3A). We also found that in undifferentiated and differentiated podocytes IQGAP1 interacted with MAGI-1 and CD2AP, proteins already known as interacting with nephrin [27], [28] (Figures 3B and 3C). Interactions between IQGAP1 and podocin or NCK1/2 were detected in podocytes (Figure 3D and 3E). We also found that IQGAP1 interacted with α-actinin-4 and β-catenin (Figure 3F and 3G), in undifferentiated and differentiated podocytes. Finally, we observed an interaction between IQGAP1 and podocalyxin (Figure 3H), an apical transmembrane protein linked to actin cytoskeleton via ezrin, expressed in podocyte foot processes and involved in cell polarity through an indirect interaction with focal membrane basal adherences [29], [30], [31].

**Figure 6:**
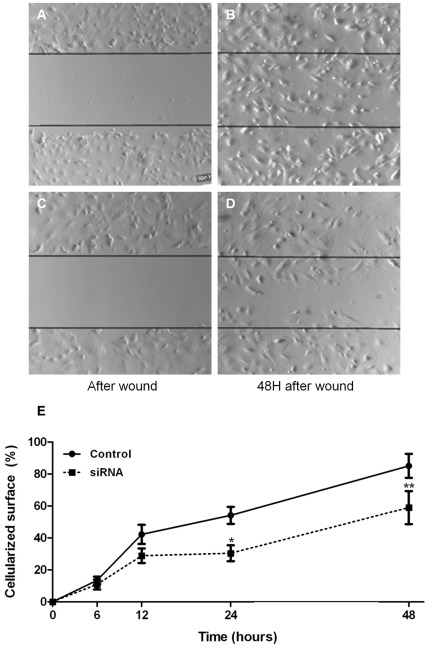
Involvement of IQGAP1 in podocyte migration. To assess the role of IQGAP1 in cell migration, migrations assay were performed with control cells (Figure 6A and B) compared to siRNA IQGAP1 transfected cells (Figure 6C and D). Cell migration was evaluated during 48 hours with sequential images acquisitions. Wound closure was expressed as the percentage of cellularized surface (data are representative of 5 independent experiments, Figure 6E). Control: control cells; siRNA: siRNA IQGAP1 transfected podocytes.

### Cellular Localization of Contacts Between IQGAP1 and Nephrin, MAGI-1, CD2AP, Podocin, NCK and Podocalyxin

In order to confirm immunoprecipitation experiments and to study protein interactions within cells, we performed In situ Proximity Ligation assays, a distance between two proteins of less than 30–40 nm giving a fluorescent dot signal [32]. To show the specificity of the interaction, we used podocytes where IQGAP1 expression was down-regulated by small interfering RNA (siRNA) (see Table 1). First, we analyzed IQGAP1 protein in cells treated or not with siRNA by Western blot. In cells transfected with siRNA IQGAP1, IQGAP1 was down-regulated by 75% as compared with the untransfected podocytes (UT) or with siRNA Luc (control, Luciferase) transfected podocytes (Figure 4A). siRNA IQGAP1 transfection induced a cell shape change with actin fibers disruption (F-actin immunofluorescence, Figure 4B). The In situ Proximity Ligation assay confirmed interactions between IQGAP1 and proteins of the slit diaphragm complex, nephrin, MAGI-1, CD2AP, podocin and NCK1/2. Moreover, there was also an interaction between IQGAP1 and the apical transmembrane protein podocalyxin (Figure 5).

**Figure 7:**
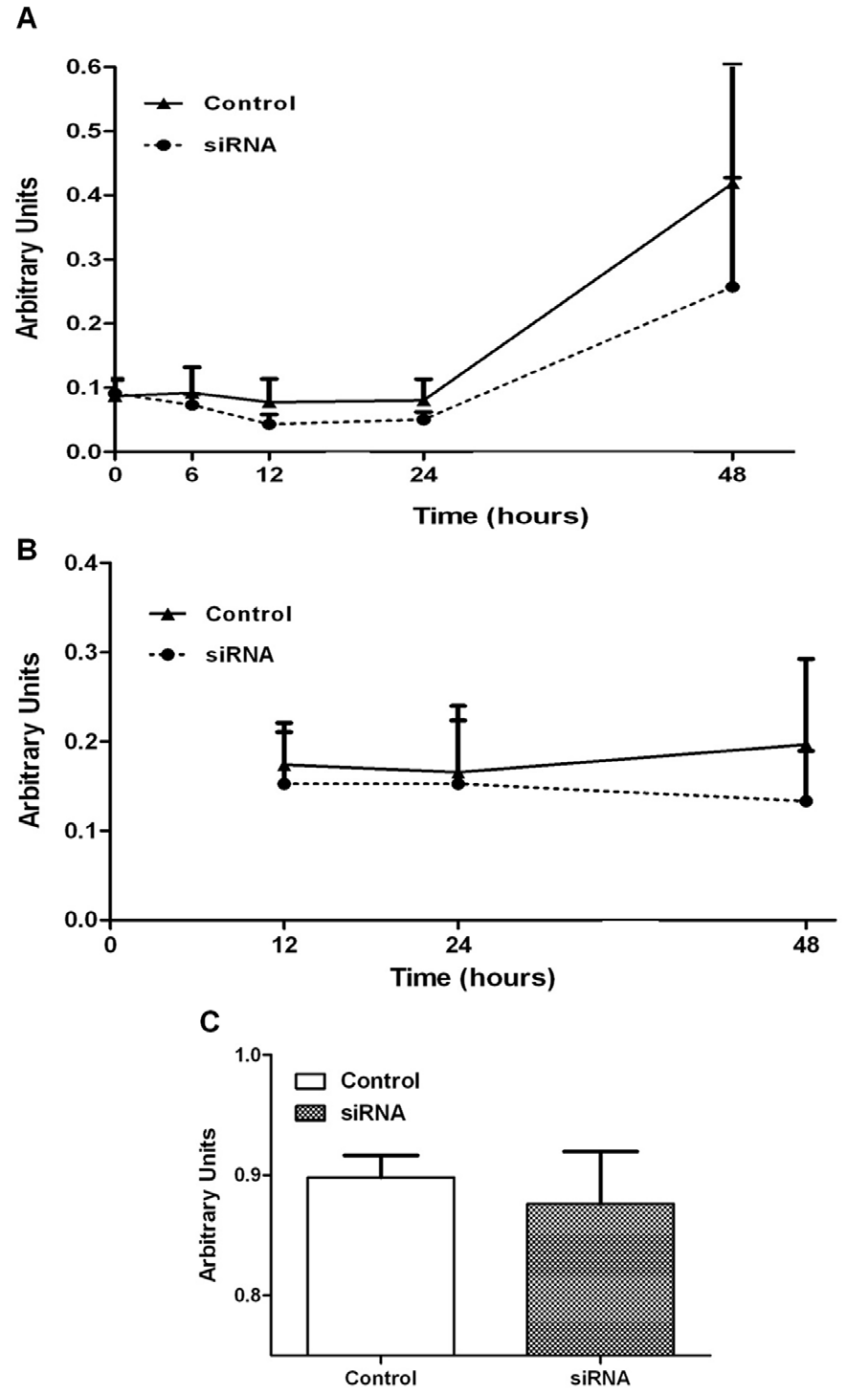
Involvement of IQGAP1 in podocyte proliferation, viability and adhesion. Influence of down regulation of IQGAP1 on cell proliferation and viability (Figure 7A and 7B, n = 5, Two-way repeated measurements ANOVA). Cell survival was not altered by transfection. Increase of cytotoxicity was not the course of the migration defect. Podocyte adhesion after down regulation of IQGAP1 (Figure 7C, n = 3, Wilcoxon test). Adhesive properties of podocytes were conserved after siRNA IQGAP1 transfection. Control: control cells; siRNA: siRNA IQGAP1 transfected podocytes.

**Figure 8:**
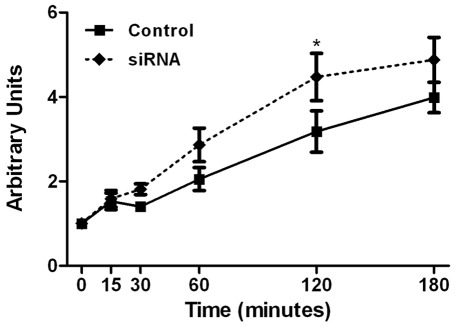
Permeability assay. Permeability assays were performed by quantification of FITC-labelled dextran (70 kDa) passage through a podocyte layer seeded in a Transwell®. The fluorescence was measured at sequential times with a Wallac® plate reader. The down-regulation of IQGAP1 expression in podocytes by siRNA induced an increase of the dextran flux across the podocyte layer (data are representative of 4 independent experiments). Control: control cells; siRNA: siRNA IQGAP1 transfected podocytes.

**Figure 9:**
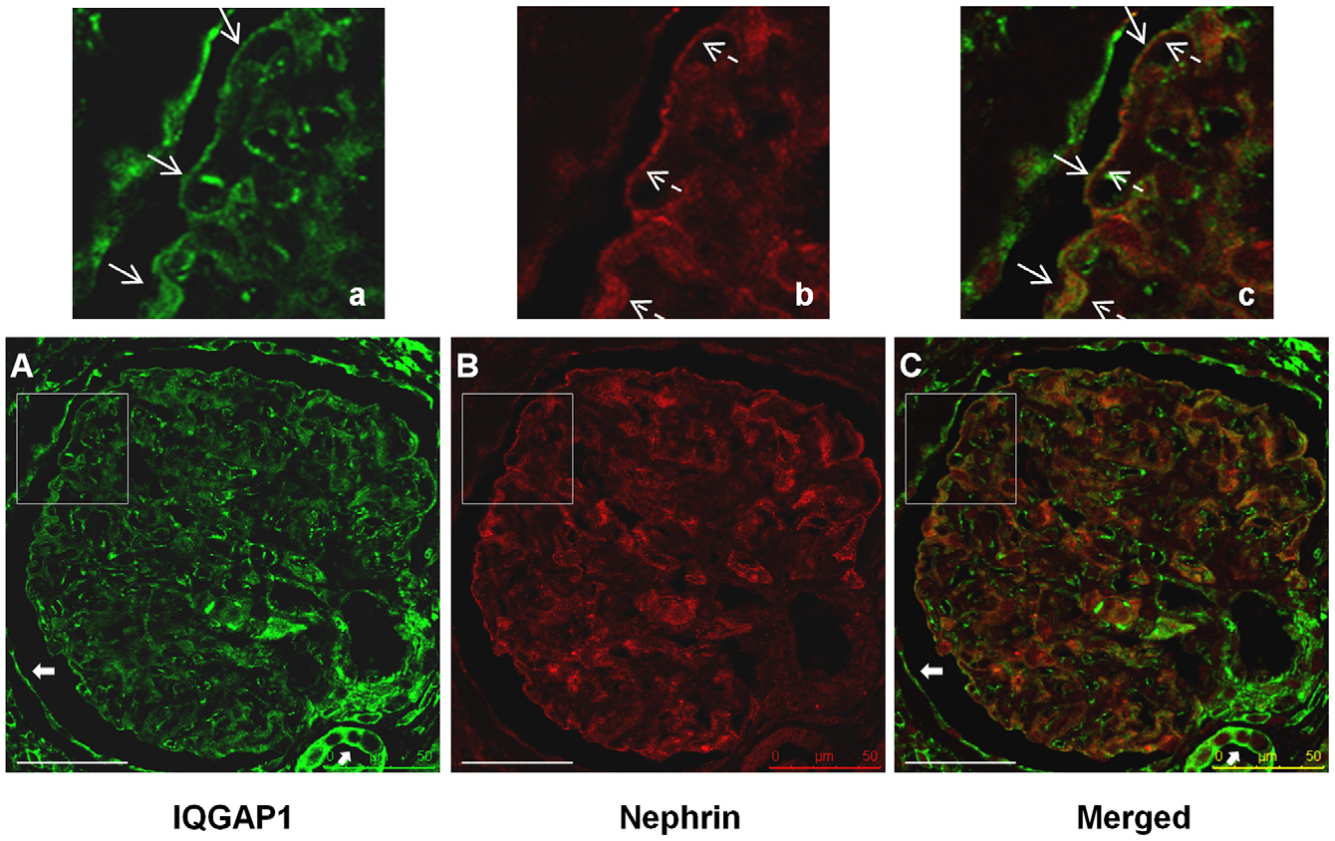
IQGAP1 glomerular expression and localization in normal kidney tissue. In normal glomeruli, IQGAP1 labelling (green, Panel A, a) was continuous, corresponding to podocytes (plain and fine arrows →), as was nephrin staining (red, Panel B and b, dashed arrow ? ). The expression in parietal epithelial, endothelial and distal tubular cells in normal glomeruli is shown respectively by bold arrows in Panel A and C. Panel C showed the co-expression of IQGAP1 and nephrin. Scale bars, 50 µm. Panels a, b and c are enlarged images of the boxed areas in panels A, B and C respectively. Results shown are representative of paraffin kidney sections from 7 normal kidneys (Results of the 6 other kidneys are available in Figure S5). Scale bar, 50 µm. Analysis was performed with a confocal microscope (Leica). Microscope sections, 0.5 µm. Magnification, X40.

### IQGAP1 Involvement in Podocyte Characteristics

In order to understand the potential role of IQGAP1 in podocyte physiology, different functional experiments were performed on cells untransfected or transfected with different siRNA (Table 1). Two controls were used as previously: untransfected cells and cells transfected with siRNA Luc. Cells were used 96 h after transfection. In the migration assay, wounds in the cell layer were reduced by 85±7.5% (median ± SEM) in control cells after 48 hours (Figure 6A and 6B). IQGAP1 silencing significantly reduced podocyte migration (Figure 6C and 6D), after 24 and 48 hours (30±4.95%, p<0.05 and 59±10%, p<0.01 respectively, two-way repeated measurements ANOVA, Figure 6E). Analysis on shorter time periods, 6 and 12 hours, showed no difference between control and siRNA IQGAP1 transfected podocytes. The inhibition of IQGAP1 expression was conserved during the migration assay (data not shown).

**Figure 10:**
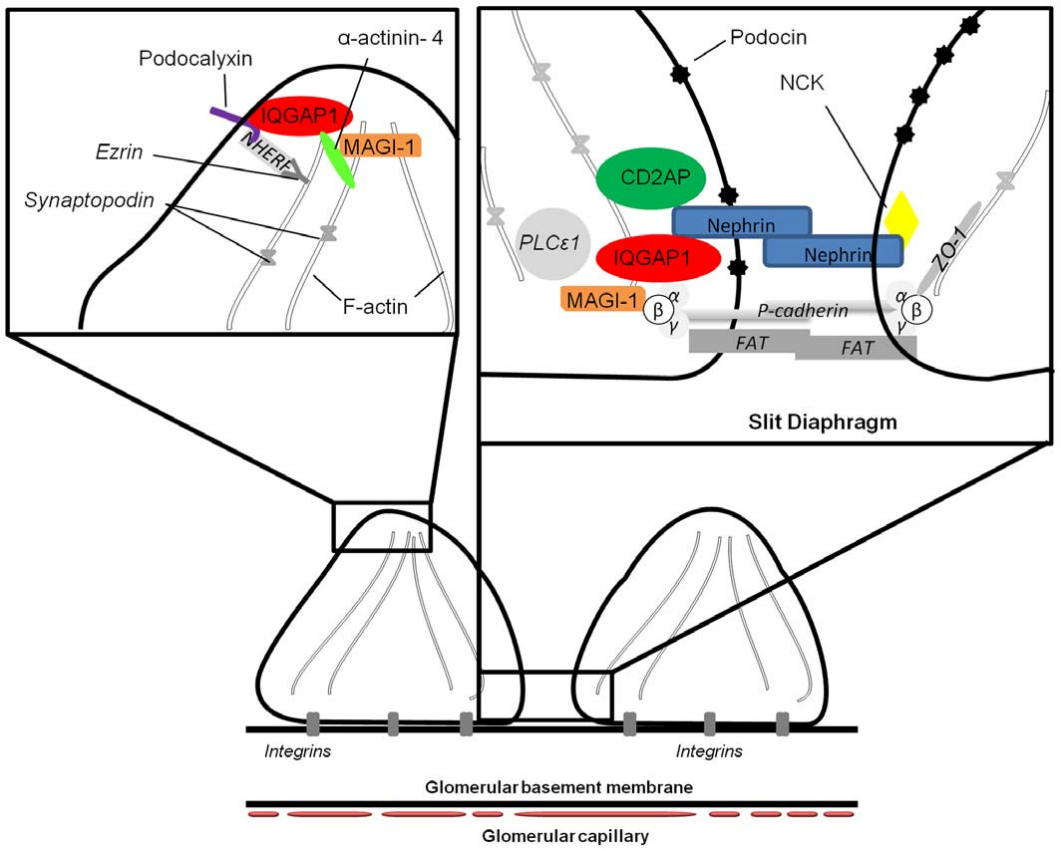
Schematic representation of the podocyte foot processes with putative IQGAP1 localization and interactions. Bottom: schematic representation of two pedicels, joined by the slit diaphragm. Top: two enlargements are depicted representing possible IQGAP1 interactions at the slit diaphragm (right) and the podocyte apical (left) regions. Proteins highlighted in grey and italics correspond to proteins not explored in this study. With the exception of nephrin, direct or indirect interactions between IQGAP1 and its partners remain to be demonstrated.

No significant difference was detected between control (untransfected cells or siRNA Luc) and siRNA IQGAP1 transfected podocytes in terms of cell proliferation and cytotoxicity or adhesion at each time point of the migration assays (Figure 7A to 7C). Impairment of podocyte migration, in siRNA IQGAP1 transfected cells, is not due to an inhibitory effect on cell proliferation, cytotoxicity or adhesion. These data were confirmed with other tests, Live-Dead test, Dead Cell Apoptosis Kit with Alexa Fluor 488 annexin V and propidium iodide in flow cytometry experiments (data not shown). Focal adhesion kinase (FAK) immunofluorescence did not show difference in focal adhesion sites between control and siRNA IQGAP1 transfected cells (Figure S3).

**Table 2:** Characteristics of primary antibodies used in the study.

Antibodies	Source	Applications (Dilution)	Origin
IQGAP1	Mouse mAb, clone AF4	IF (1/100)	Upstate
	Rabbit pAb, H-109	IH (1/500), IF (1/100)	Santa Cruz
		WB (1/1000), IP (2 µg/ml)	
Nephrin	Rabbit pAb, H-300	IF (1/50), WB (1/250)	Santa Cruz
		IP (3 µg/ml), IH (1/50)	
	Guinea pig	IH (1/50)	Acris
MAGI-1	Rabbit pAb, H-70	IF (1/100), WB (1/250), IP (3 µg/ml)	Santa Cruz
CD2AP	Rabbit pAb, H-290	IF (1/100), WB (1/250), IP (3 µg/ml)	Santa Cruz
β-actin	Mouse mAb, clone C4	IF (1/100), WB (1/10000)	BD Bioscience
β-catenin	Mouse mAb, clone 14	IF (1/100), WB (1/1000)	BD Bioscience
	Rabbit mAb	IF (1/100), WB (1/1000), IP (3 µg/ml)	Cell signaling
α-actinin-4	Rabbit pAb	IF (1/100), WB (1/250), IP (3 µg/ml)	Alexis
Podocalyxin	Mouse mAb	IF (1/100), WB (1/250), IP (3 µg/ml)	Gift Pr Ronco
NCK 1-2	Rabbit pAb, C-19	IF (1/100), WB (1/250), IP (3 µg/ml)	Santa Cruz
	Rabbit mAb	IF (1/100)	Cell signaling
Podocin	Rabbit pAb	IF (1/100), WB (1/250), IP (3 µg/ml)	Santa Cruz
TTP	Rabbit pAb, H-120	IP (2 µg/ml)	Santa Cruz

(Abbreviations: mAb = monoclonal Antibody, pAb = polyclonal Antibody, IH = immunohistochemistry, IF = Immunofluorescence, WB = Western blot, IP = Immunoprecipitation).

A permeability assay was performed in differentiated cells transfected for 96 h. A significant increase in the podocyte layer permeability to 70 kDa FITC-dextran was observed at 120 minutes in siRNA IQGAP1 transfected cells compared to control cells (3.1±0.4 vs 4.5±0.5 respectively, p<0.05, two-way repeated measurements ANOVA, Figure 8).

**Table 3:** Characteristics of the secondary antibodies used in the study.

Antibodies	Conjugate to	Applications	Origin
Goat anti rabbit	IR Dye 680	WB	LI-COR, Odyssey
	IR Dye 800	WB	LI-COR, Odyssey
Goat anti mouse	IR Dye 680	WB	LI-COR, Odyssey
	IR Dye 800	WB	LI-COR, Odyssey
Goat anti rabbit	Alexa Fluor 594	IF, IH	Molecular Probes
	Alexa Fluor 488	IF, IH	Molecular Probes
Goat anti mouse	Alexa Fluor 488	IF	Molecular Probes
	Alexa Fluor 594	IF	Molecular Probes
Goat anti guinea pig	Alexa fluor 568	IH	Molecular Probes
Swine anti rabbit	HRP	WB	Dako

(Abbreviations: IH = immunohistochemistry, IF = Immunofluorescence, WB = Western blot, IP = Immunoprecipitation).

### IQGAP1 Expression in Normal Glomeruli

In order to study IQGAP1 expression *in vivo* in human podocytes, IQGAP1 immunohistochemistry experiments were performed on normal kidney sections (biopsies obtained during kidney transplantation from living donors, n = 7). To discriminate between podocytes and other components of glomeruli, nephrin was used as a specific marker for podocytes (additional DAPI nuclear staining and nephrin staining data are available in Figure S4A and S4B). On normal kidney sections, IQGAP1 labelling was found in podocyte processes and cell bodies (Figure 9A, C, a and c, plain and fine arrows). IQGAP1 was expressed in glomerular parietal epithelial cells (bold arrows in Figure 9A and C). IQGAP1 was also expressed in endothelial [33] and distal tubular cells (bold arrows in Figure 9A and C) but was absent in proximal tubular cells [34]. Nephrin was expressed in podocytes (Figure 9B and C, insert b and c, dash arrows). An inconstant colocalization with IQGAP1 was observed in podocytes (Figure 9C and c, middle and lower plain and dashed arrows). Indeed, in some glomerular areas, nephrin and IQGAP1 appeared juxtaposed at the same podocyte structure rather than directly colocalized (Figure 9c, top arrows). Similar results were obtained with additional human kidney sections (Figure S5).

## Discussion

In the present report, we demonstrate that apart from being associated to nephrin in podocytes [7], [8], IQGAP1 is associated to other proteins of the slit-diaphragm complex and of the cytoskeleton. Furthermore, IQGAP1 is involved in functional properties of podocytes such as permeability as a cell layer, and migration.

IQGAP1 expression increased from undifferentiated to differentiated stages, as shown for other slit diaphragm proteins such as podocin and nephrin [25]. The down-regulation of IQGAP1 expression in podocytes induces a disorganization of actin cytoskeleton together with a disruption of actin fibers and cell shape changes. IQGAP1 is crucial in the cytoskeleton organization: several studies reported an inhibition of the actin meshwork formation, and an alteration in cell adhesion and migration following the down regulation of IQGAP1 expression [17], [21], [23]. Therefore, IQGAP1 may be important in the major cytoskeletal rearrangement occurring during podocyte differentiation and acquisition of the mature podocyte phenotype [8], [35].

Our study first suggests that IQGAP1 is an integral partner of the nephrin complex and may connect the cytoskeleton with the slit diaphragm complex, through its interaction with members of this complex and cytoskeletal proteins. IQGAP1 also colocalized with β-catenin and MAGI-1, at cell contacts. Depending on the cell type, IQGAP1 can increase or decrease the strength of the cell-cell junction through binding to β-catenin [18], [19], a cadherin adaptator protein. Thanks to its interaction with β-catenin, IQGAP1 may be a modulator of the strength of the interaction between podocytes. Indeed, podocyte junctions depend on P-cadherin and its interaction with catenins [36]. Finally, we also show that IQGAP1 interacted with podocalyxin, a protein expressed at the apical domain of podocytes, early expressed during the glomerular development and crucial in determining the cell polarity [29], [30], [31]. IQGAP1 is expressed at different sites in the cell and particularly at the apical area, where its association with podocalyxin may contribute to associate podocalyxin to podocyte cytoskeleton. Our study does not allow distinguishing whether IQGAP1 interactions with these proteins are direct or indirect. IQGAP1 was shown to bind directly β-catenin [18] and F-actin [37] in various cell lines. In podocytes, we may speculate that there is a direct interaction between these proteins and IQGAP1. Further experiments are needed to answer these questions.

Among the three podocyte domains usually described (apical, basal domains and the slit diaphragm) [38], our work shows that IQGAP1 is expressed in two areas, the slit diaphragm and the apical domain. Following published data [1], [2], [3], [4], [5], [6], [7], [8] and results from our study, a schematic representation of foot podocyte processes summarizing IQGAP1 localization and potential interactions in podocytes is proposed in Figure 10.

Functional experiments showed that IQGAP1 has functional importance in podocyte physiology. First, IQGAP1 was found to be involved in podocyte migration. Described in other cell types, the silencing of IQGAP1 expression modifies its interactions with proteins as APC, ARP 2/3, N-WASP or CLIP170 and induces actin cytoskeleton disorganization with microtubule stabilization defect. This impairment results in trafficking abnormalities of tubulin units, inhibition of the actin meshwork formation at the leading edge front and then cell motility defect [16], [17], [21], [22], [23], [39]. Podocytes are motile cells around the glomerular basement membrane [40]. An alteration of podocyte motility is associated to proteinuria [41]. For this reason, the putative role of IQGAP1 in the motility of podocytes was important to clarify. The lack of a significant effect of IQGAP1 inhibition by RNA interference on podocyte adhesion to culture plates after silencing suggests that IQGAP1 is dispensable for podocyte adhesion to culture plates. This may due to redundancy with other IQGAP family member, such as IQGAP2 also involved in cytoskeleton regulation [22] or redundancy with other adhesion mechanisms to culture plates. Our results also show that IQGAP1 silencing induces a loss of podocyte layer permeability. IQGAP1 involvement in the control of endothelial cell permeability has been reported [42]. This modification may be due to podocyte shape change linked to IQGAP1 contribution to the actin cytoskeleton remodelling. Podocyte cytoskeleton remodelling is critical in foot processes effacement and slit diaphragm disruption [5], [43]. A defect of IQGAP1 expression may impair interactions between the slit diaphragm and cytoskeleton, first step of foot processes effacement. Then, IQGAP1 may be important in the glomerular barrier integrity and in glomerular disease pathophysiology, through its contribution to key podocyte characteristics (podocyte plasticity, migration and permeability). Studies on tissue sections from patients suffering from minimal change disease, focal and segmental glomerulosclerosis or diabetes may be interesting to clarify the putative role of IQGAP1 in pathogenesis. Our preliminary results of IQGAP1 expression on pathological human tissue sections are too variable from patient to patient to conclude (data not shown). However, unlike other proteins such as nephrin, podocin, PLCε1, CD2AP, α-actinin-4, or NCK [1], [3], [4], [5], [6] which are crucial in glomeruli formation and in the development of congenital nephropathies, mutant IQGAP1 mice present only gastric hyperplasia without renal phenotype including proteinuria [44]. IQGAP family members, IQGAP1, IQGAP2 and IQGAP3, present a high degree of homology. In mutant IQGAP1 mice, a functional redundancy of IQGAP2 (expression level of IQGAP2 in IQGAP1 deficient mice is not altered) or overexpression of IQGAP3 could blunt the renal effects of IQGAP1 expression and/or function alterations.

In conclusion, IQGAP1, a scaffold protein, is expressed by human podocytes in culture, where it interacts with several proteins of the slit diaphragm nephrin, MAGI-1, CD2AP, podocin, NCK1/2, with proteins of the actin cytoskeleton, α-actinin-4 and β-actin, with β-catenin, and with the apical protein podocalyxin. Down-regulation of IQGAP1 expression diminished podocyte migration properties, and increased podocyte layer permeability. Through its connections with foot process cytoskeleton and slit diaphragm proteins, IQGAP1 may control key aspects of podocyte biology and may be important in the pathophysiology of glomerular diseases.

## Methods

### Cell Culture

The podocyte cell line has been previously described [25], [26]. The cells were obtained from human nephrectomy specimen without glomerular disease and conditionally immortalized by the use of the temperature-sensitive large T antigen-SV40 transgene. These cells were fully differentiated after a switch from 33°C (permissive, proliferative temperature) to 37°C (non permissive, differentiating temperature) for 16 days. Cells were cultured in RPMI 1640 medium supplemented with 10% fetal calf serum (FCS), insulin (10 µg/ml, Sigma-Aldrich®, Lyon, France), transferrin (5,5 µg/ml, Sigma-Aldrich®), selenium (5 ng/ml, Sigma-Aldrich®) and Penicillin-Streptomycin 1X (100X, Invitrogen, Cergy Pontoise, France). Flasks were coated with collagen I rat rail (67 µg/ml, Sigma-Aldrich®).

### Antibodies

Primary and secondary antibodies used in this study are listed in Tables 2 and 3.

### Protein Extraction

Cells were washed in phosphate buffer saline (PBS) and scraped. After centrifugation at 1,200 g for 5 min, the cell pellet was resuspended in lysis buffer (10 mM Tris HCl pH 7.5, 5 mM EDTA, 80 mM NaCl, 50 mM NaF, 1% Triton X100) with 5µl/ml protease inhibitor (Sigma-Aldrich®) and 50 UI/ml desoxyribonuclease I (Invitrogen). The suspension was mixed for 1 h at 4°C and then centrifuged at 15,000 g, for 15 minutes, at 4°C. The supernatant was analyzed.

For Western blot analysis, human kidney lysates from nephrectomy specimen without nephropathy were used as control.

Protein quantification was performed using a Bradford assay (Biorad, Marnes-la-Coquette, France) using Bovine Serum Albumin (BSA) as standard.

### Western Blotting

SDS-PAGE with 6 or 10% acrylamide was performed. A total of 20 µg of extracted protein were loaded in each lane. After protein transfer on nitrocellulose, membranes were blocked with TBST (10 mM Tris HCl pH 7.5, 140 mM NaCl, 0.1% Triton X100) and 5% BSA, and then incubated with primary antibodies overnight, at 4°C (Table 2). After several washes with TBST, the membrane was incubated for 1 h at room temperature with the appropriate secondary antibodies conjugated to an IR Dye at 1/5000 dilution (Table 3). After final washes, the membranes were visualised using an Odyssey fluorescence imager (LI-COR Biosciences, Courtaboeuf, France). In some experiments a swine anti rabbit immunoglobulin conjugated to horse-radish peroxydase (HRP) was used as secondary antibody. The detection was performed using an enhanced chemiluminescent substrate (Pierce, Brebières, France) and an Odyssey fluorescence imager or film. Densitometry of signals was performed using the Quantity One software (Bio-Rad).

### Immunoprecipitation

500 µg of protein extracts were precleared with 50 µl of protein-G Sepharose beads (Sigma-Aldrich®) for 1 h at 4°C. Then, podocyte lysates were incubated overnight at 4°C with 2 µg/ml of anti-IQGAP1 polyclonal antibody or 3 µg/ml of the indicated antibodies (for the reverse IP). 40 µl of protein-G Sepharose beads were added for 2 h at 4°C. Beads were washed extensively with cold lysis buffer. Samples boiled in SDS PAGE sample buffer were then used for Western blotting experiments. IP controls were realized, in same conditions, with an irrelevant polyclonal antibody (rabbit polyclonal anti Tristetraprolin, a protein not interacting with IQGAP1 and proteins analyzed in this work).

### Immunofluorescence

Cells cultivated on coverslips were fixed in 3% paraformaldehyde in Cytoskeleton Buffer (10 mM MES pH 6.1, 150 mM NaCl, 5 mM EGTA, 5 mM MgCl_2_, 5 mM glucose) for 10 min. After several washes in TBS (TBST without Triton X100), cells were permeabilized 30 sec in 0.1% Triton X100, washed in TBS and blocked with TBS containing 1% Bovine Serum Albumin (BSA) and 1% Fetal Calf Serum (FCS). Cells were incubated with the appropriate primary antibody, at a 1/100 dilution (except for nephrin at a 1/50 dilution) (Table 2) in blocking buffer, for 2 h, at room temperature. After several washes, cells were incubated with the appropriate secondary antibody or Alexa Fluor® 594 phalloïdin (Molecular Probes®, Invitrogen) at a 1/200 dilution, for 1 h at room temperature (Table 3). After several washes, nuclei were labelled with DAPI solution (dilution 1/1000) for 1 min. Then, coverslips were mounted on slides using Fluoromount-G (Southern Biotech, USA). Labelling were analysed using a confocal microscope (Leica).

### Small Interfering RNA (siRNA) Experiments

400,000 podocytes were seeded on a 6 cm culture dish. Differentiated podocytes were double transfected with siRNA IQGAP1using the lipofectant RNAiMax (Invitrogen): briefly, 7.5 µl of the lipofectant were mixed with 80 pmol of siRNA in 1 ml of Opti MEM. The mix was added to 4 ml of complete media without antibiotics to the dishes. Cells were incubated overnight at 37°C and 5% CO_2_. The following day, the same concentrations of lipofectant and siRNA in 1 ml of Opti MEM were added to the dishes. Cells were used between 72 h and 96 h after the second transfection. The sequences of the siRNA targeting IQGAP1 (siRNA 672 or 3014) and the luciferase (control, siRNA Luc) are reported in Table 1.

### In Situ Proximity Ligation Assay

Protein interactions in podocytes were studied using an In situ Proximity Ligation assay Kit (Duolink®) from Olink Bioscience, Finland [32]. Cells transfected with the siRNA Luc or 672 (Table 1) and cultured on coverslips were fixed, permeabilized and incubated with primary antibodies such as in immunofluorescence assays. Next, the In situ Proximity Ligation assay was used as recommended by the manufacturer. Briefly, cells were incubated with secondary antibodies with attached nucleotides. If nucleotides were closed (less than 30–40 nm), the addition of two more nucleotides and the ligation resulted in the formation of a circular DNA strand. After amplification of the DNA circle and hybridization of fluorescence (563 nm) labelled complementary oligonucleotide detection probes, protein interactions were visualized with a confocal microscope (Leica) as a red dot.

### Migration Assay

96 h after transfection (siRNA Luc or 3014 sequences), a migration assay was performed on adherent and fully differentiated cells (14 days after switching from 33°C to 37°C) in a 6 well plate. For the migration assay, the medium was changed (for low serum medium 1% fetal calf serum). 2 wounds per well were realized with a sterile tip. Pictures were sequentially taken just before and after the scrap (T0), 6, 12, 24, 48 hours with a light microscope. Cells having migrated inside the wound were counted.

### Permeability Assay

96 h after cell transfection (siRNA Luc or 3014 sequences), permeability assay was assessed by measuring the passage of FITC-labelled dextran (Molecular weight: 70 kDa, Invitrogen®) across a Transwell® seeded with podocytes 16 days before (2 days of proliferation and 14 days of differentiation). Cells were starved during 1 hour (medium replacement was performed in wells and Transwells®). Then, 100 µl of Transwell® and well medium was removed. The volumes were replaced with 100 µl of serum free medium containing 1 mg/ml of FITC-labelled dextran in the Transwell® and with 100 µl of serum free medium containing 1 mg/ml of unlabelled dextran in the well. At 1, 2 and 3 hours, 100 µl of medium was removed from each well and replaced with 100 µl of serum free medium containing 100 µg/ml of unlabelled dextran. Each sample was transferred in a 96 wells plate for fluorescence measurement.

### Cytotoxicity Assay

After 96 h of transfection (siRNA Luc or 3014), cytotoxicity was assayed on differentiated cells. 3-(4,5-Dimethylthiazol-2-yl)-2,5-diphenyltetrazolium bromide (MTT) was used at a 1/5 dilution. Medium was removed. Cells were washed with Hank’s medium and incubated with MTT solution for 3 h at 37°C. MTT solution was removed and replaced by cell lysis buffer: DMSO (Dimethylsulfoxyde). Samples were then transfered in a 96 well plate for spectrophotometer measuring.

### Proliferation Assay

After 96 h of transfection (siRNA Luc or 3014 sequences), proliferation assayed on differentiated cells. Neutral red was used at a 1/80 dilution in IMDM with 10% FCS. Medium was removed. Cells were washed with Hank’s medium and incubated with neutral red solution for 3 h at 37°C. Solution was removed and replaced by cell lysis buffer: acetic acid (1%) with ethanol (50%). Samples were then transfered in a 96 well plate for spectrophotometer measuring.

### Quantification of Adherent Cells

To appreciate the proportion of adherent cells, Sulforhodamine B assay was done. We used the In vitro toxicology assay kit Sulforhodamine B Based (Sigma-Aldrich®) and followed the manufacturer procedure.

### Immunohistofluorescence

10 µm-thick kidney sections were deparaffined, hydrated and treated for 30 min at 98°C with 10 mM citrate buffer, pH 6 for antigen retrieval. Sections were washed in PBS and incubated for 30 min with 3% BSA in PBS to block non specific binding. Then sections were incubated overnight at 4°C with primary antibodies against IQGAP1 and nephrin at a 1/50 dilution in PBS (Table 2). After several washes in PBS, sections were incubated for 1 h at room temperature with appropriate secondary antibodies at a 1/200 dilution in PBS (Table 3). After several washes in PBS, nuclei were labelled with DAPI (dilution 1/1000 in PBS). Then sections were mounted with Fluoromount-G (Southern Biotech, USA) and analysed using a confocal microscope (Leica).

### Statistical Analysis

Statistical significance was assessed either by parametric or non-parametric tests, as appropriate with GraphPad® Software (La Jolla, CA). Statistical significance was defined as p<0.05.

## Supporting Information

Figure S1 **Morphology of human cultured podocytes and expression of nephrin, a podocyte specific marker.** (A) The morphology of human cultured podocytes was observed by light microscopy: undifferentiated cells (left panel-33°C) and differentiated cells (right panel-37°C). (B) Immunofluorescence of nephrin in human cultured podocytes showed a diffuse cytoplasmic expression in undifferentiated cells (left panel-33°C) and a focal membrane expression associated with a cytoplasmic labelling in differentiated cells (right panel-37°C).
(TIF)

Figure S2 **Western blot analysis of IQGAP1, nephrin, MAGI-1, CD2AP, podocin, NCK 1/2, α-actinin-4, β-catenin, podocalyxin and β-actin expression in cultured podocyte extracts.** Western blot analyses were performed on all these proteins during podocyte differentiation. Protein extracts were from undifferentiated (permissive temperature of 33°C, Day 0 (D0)) and differentiated (non-permissive temperature of 37°C, Day 16 (D16)) cultured immortalized podocytes, and a second human kidney lysate (HKL); MW: Molecular weight (kDa).
(TIF)

Figure S3 **FAK expression in podocytes.** Immunofluorescence of FAK showed a similar expression in control podocytes (A) and in siRNA IQGAP1 transfected podocytes (B). Characteristics of FAK antibody: rabbit antibody, dilution 1/100, Upstate Biotechnology, Massachusetts, USA.
(TIF)

Figure S4 **Nuclear staining in normal kidney tissue.** In conditions previously described, IQGAP1 labelling (green) and nuclear staining with DAPI was performed. Labelling is representative of normal kidney. Scale bar, 50 µm. Analysis was performed with a confocal microscope. Microscope sections, 0.5 µm. Magnification, X40. Nephrin staining in normal kidney tissue: In conditions previously described, nephrin labelling (red) was performed. In normal glomeruli, nephrin staining (red) was continuous around the glomerular basement membrane. Scale bar, 50 µm. Analysis was performed with a confocal microscope. Microscope sections, 0.5 µm. Magnification, X40.
(TIF)

Figure S5 **IQGAP1 glomerular expression and localization in normal kidney tissue.** All sections from normal kidneys (6) are figured. In normal glomeruli, IQGAP1 labelling (green) was continuous, corresponding to podocytes. IQGAP1 was also expressed in parietal epithelial, endothelial and distal tubular cells. Nuclear staining with DAPI is also represented. Immunohistochemistry experiments were performed on paraffin sections from normal kidneys (6). Analysis was performed with confocal microscopy. Microscope sections, 0.5 µm. Magnification, X40.
(TIF)
