# Supplementary figures and images for: IQGAP1 Interacts with Components of the Slit Diaphragm Complex in Podocytes and Is Involved in Podocyte Migration and Permeability In Vitro
Source: PLoS One. 2012 May 25;7(5):e37695. doi: 10.1371/journal.pone.0037695 (PMC3360763; doi:10.1371/journal.pone.0037695)

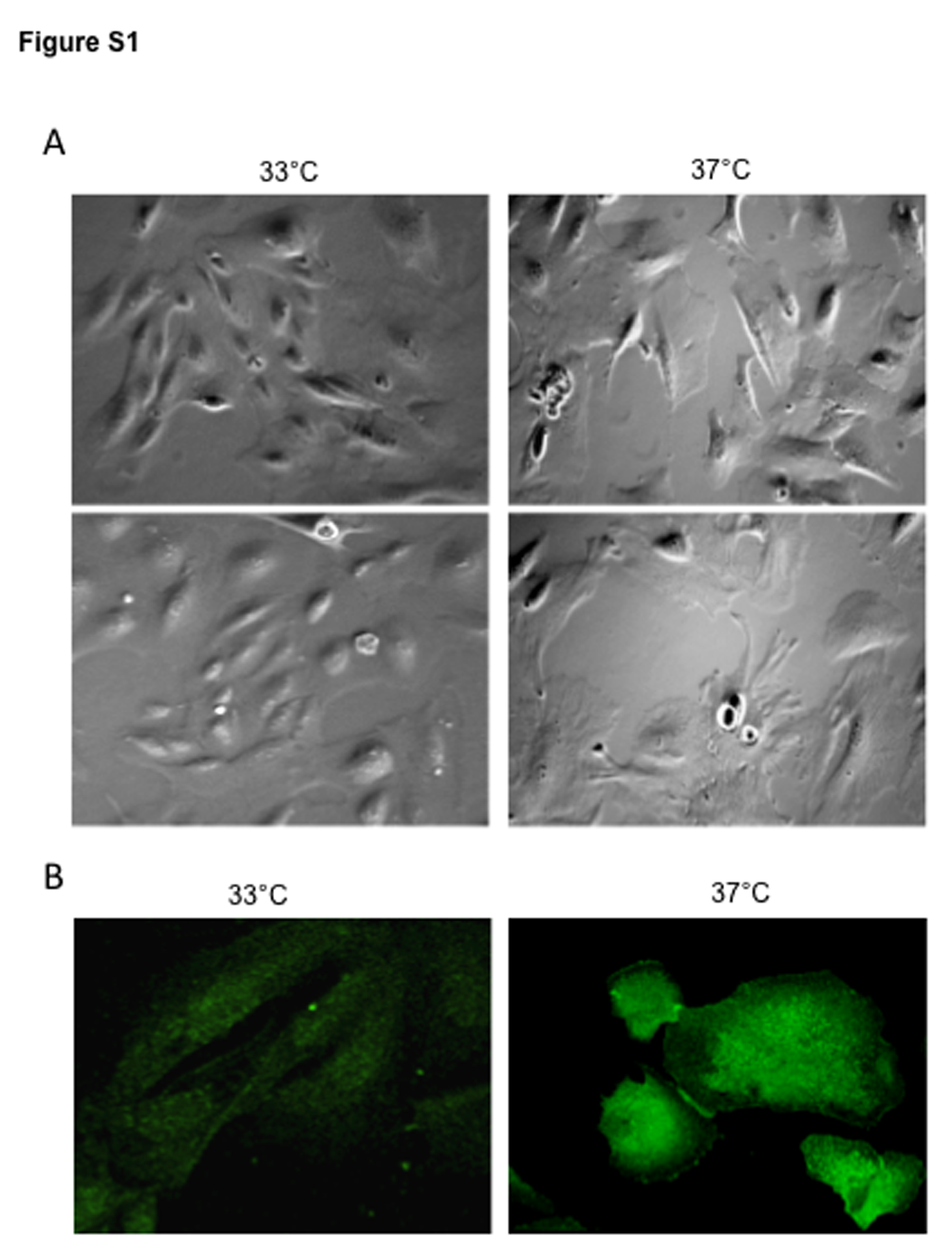

Supplement: Figure S1 — Morphology of human cultured podocytes and expression of nephrin, a podocyte specific marker. (A) The morphology of human cultured podocytes was observed by light microscopy: undifferentiated cells (left panel-33°C) and differentiated cells (right panel-37°C). (B) Immunofluorescence of nephrin in human cultured podocytes showed a diffuse cytoplasmic expression in undifferentiated cells (left panel-33°C) and a focal membrane expression associated with a cytoplasmic labelling in differentiated cells (right panel-37°C). (TIF) [file pone.0037695.s001.tif]

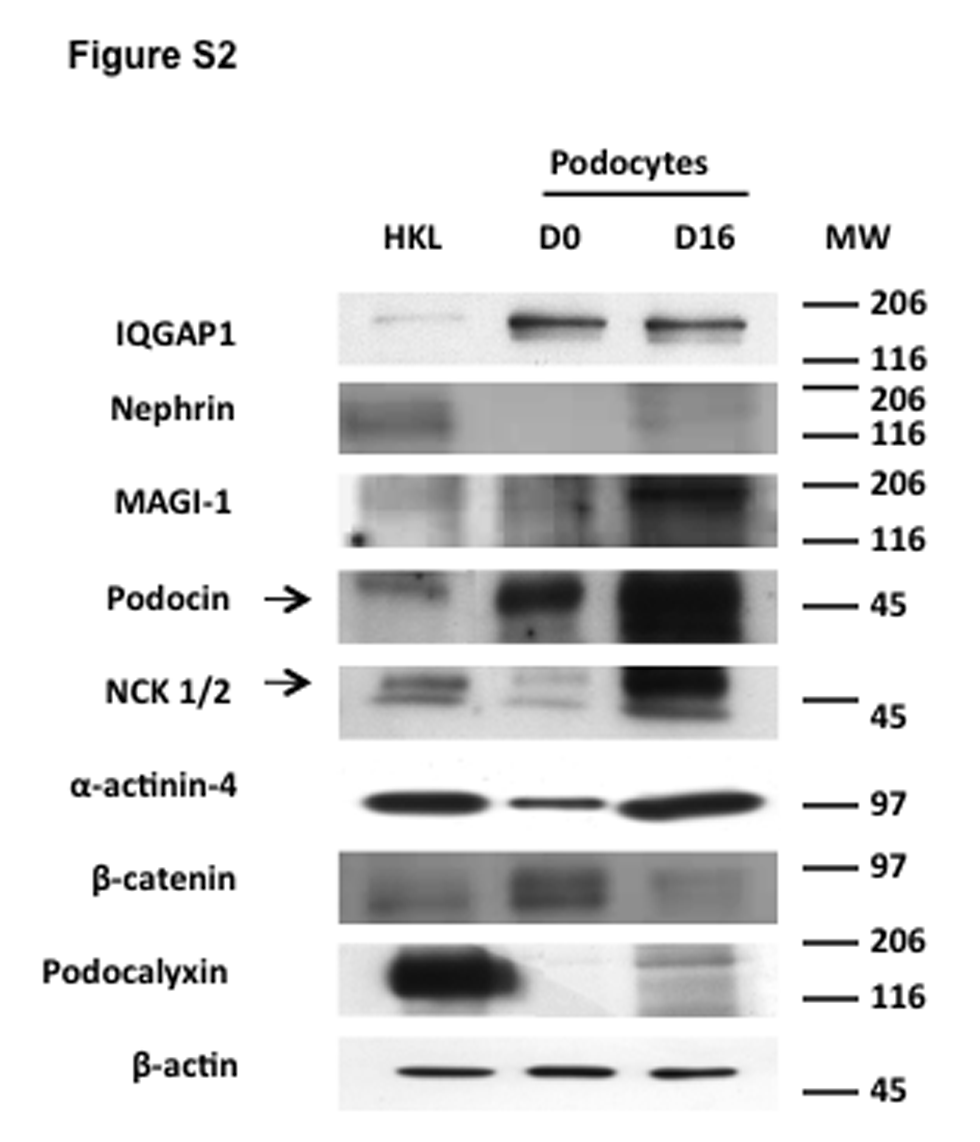

Supplement: Figure S2 — Western blot analysis of IQGAP1, nephrin, MAGI-1, CD2AP, podocin, NCK 1/2, α-actinin-4, β-catenin, podocalyxin and β-actin expression in cultured podocyte extracts. Western blot analyses were performed on all these proteins during podocyte differentiation. Protein extracts were from undifferentiated (permissive temperature of 33°C, Day 0 (D0)) and differentiated (non-permissive temperature of 37°C, Day 16 (D16)) cultured immortalized podocytes, and a second human kidney lysate (HKL); MW: Molecular weight (kDa). (TIF) [file pone.0037695.s002.tif]

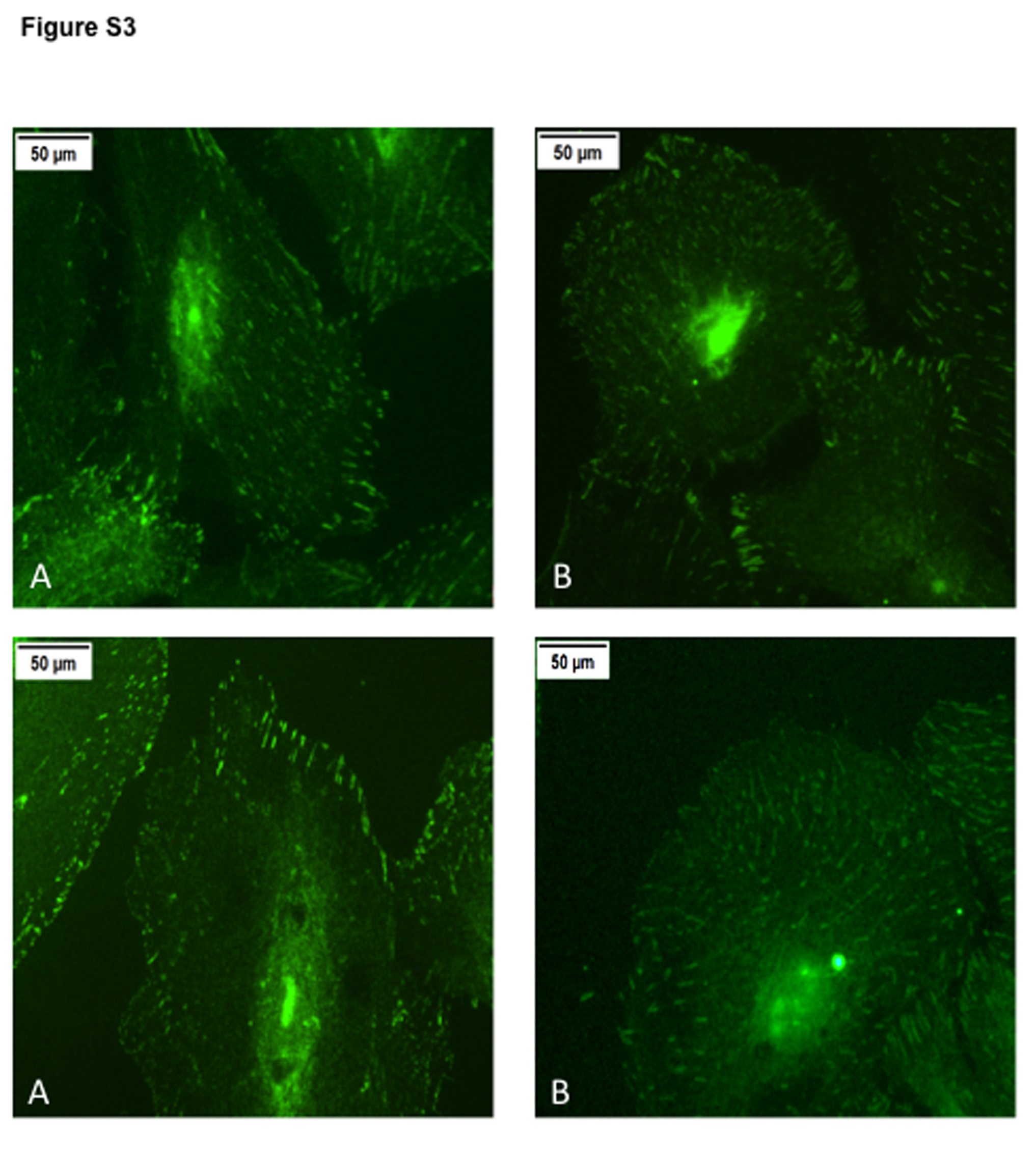

Supplement: Figure S3 — FAK expression in podocytes. Immunofluorescence of FAK showed a similar expression in control podocytes (A) and in siRNA IQGAP1 transfected podocytes (B). Characteristics of FAK antibody: rabbit antibody, dilution 1/100, Upstate Biotechnology, Massachusetts, USA. (TIF) [file pone.0037695.s003.tif]

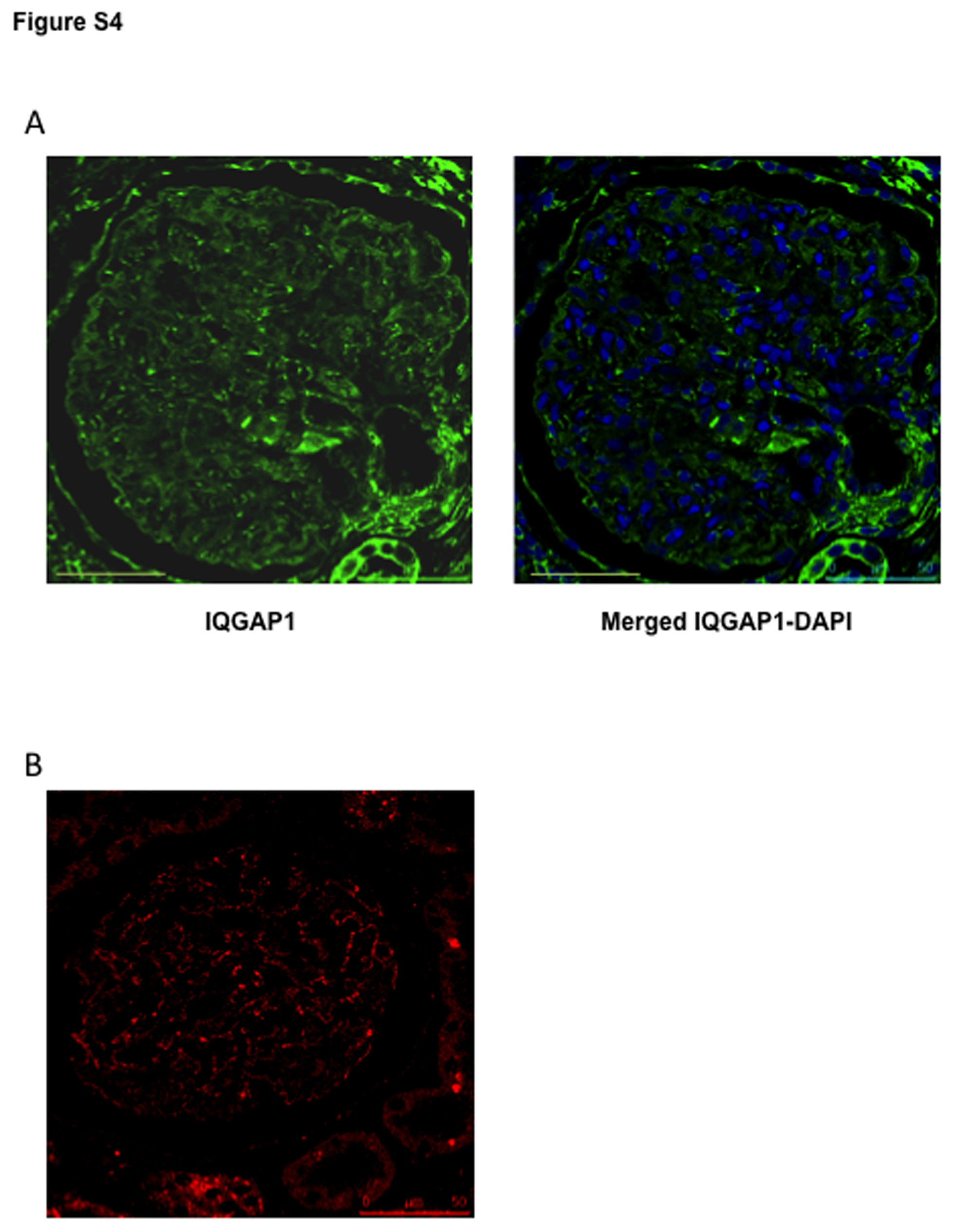

Supplement: Figure S4 — Nuclear staining in normal kidney tissue. In conditions previously described, IQGAP1 labelling (green) and nuclear staining with DAPI was performed. Labelling is representative of normal kidney. Scale bar, 50 µm. Analysis was performed with a confocal microscope. Microscope sections, 0.5 µm. Magnification, X40. Nephrin staining in normal kidney tissue: In conditions previously described, nephrin labelling (red) was performed. In normal glomeruli, nephrin staining (red) was continuous around the glomerular basement membrane. Scale bar, 50 µm. Analysis was performed with a confocal microscope. Microscope sections, 0.5 µm. Magnification, X40. (TIF) [file pone.0037695.s004.tif]

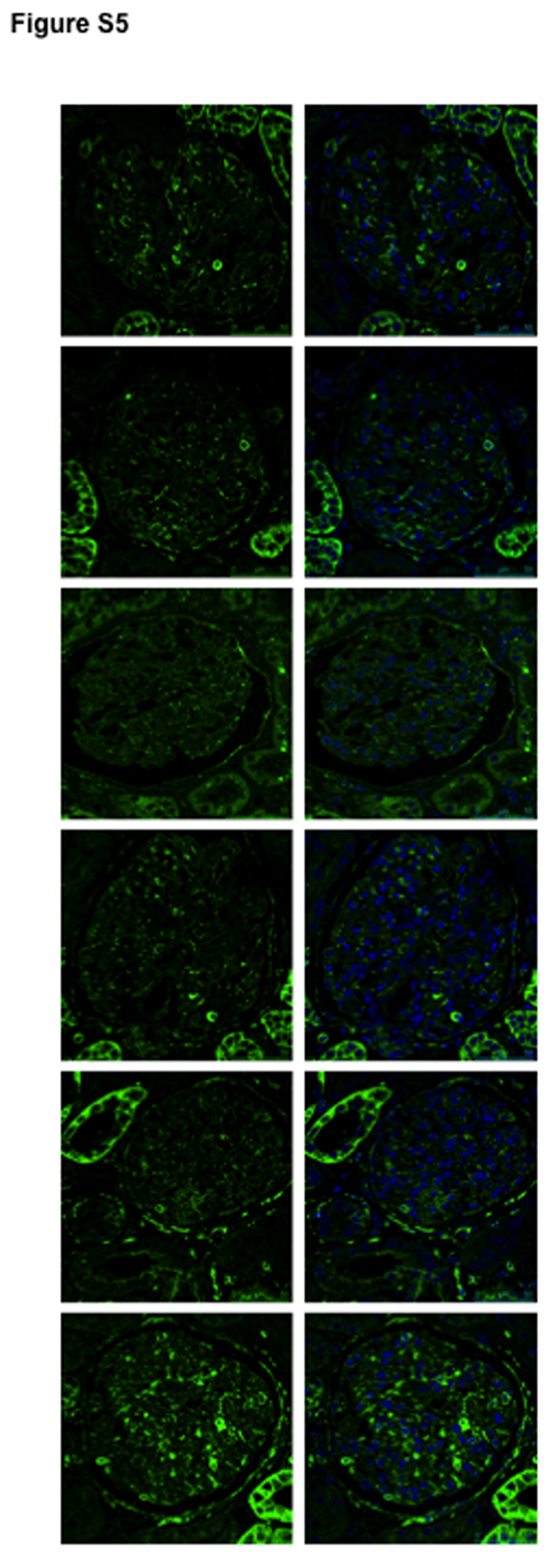

Supplement: Figure S5 — IQGAP1 glomerular expression and localization in normal kidney tissue. All sections from normal kidneys (6) are figured. In normal glomeruli, IQGAP1 labelling (green) was continuous, corresponding to podocytes. IQGAP1 was also expressed in parietal epithelial, endothelial and distal tubular cells. Nuclear staining with DAPI is also represented. Immunohistochemistry experiments were performed on paraffin sections from normal kidneys (6). Analysis was performed with confocal microscopy. Microscope sections, 0.5 µm. Magnification, X40. (TIF) [file pone.0037695.s005.tif]
